# Safety and Efficacy Data on Vaccines and Immunization to Human Papillomavirus

**DOI:** 10.3390/jcm4040614

**Published:** 2015-04-03

**Authors:** Natalie Kash, Michael A. Lee, Ramya Kollipara, Christopher Downing, Jacqueline Guidry, Stephen K. Tyring

**Affiliations:** 1 University of Texas Medical School at Houston, Houston, TX 77030, USA; E-Mail: Natalie.N.Kash@uth.tmc.edu; 2 Center for Clinical Studies, Houston, TX 77004, USA; E-Mails: ramyak48@gmail.com (R.K.); cdowning@ccstexas.com (C.D.); jguidry@ccstexas.com (J.G.); styring@ccstexas.com (S.K.T.); 3 Department of Dermatology, University of Texas Health Science Center at Houston, Houston, TX 77030, USA

**Keywords:** HPV, virology, cervical cancer, vaccination, pap smear, Gardasil, Cervarix

## Abstract

Since the discovery of the causal association between human papillomavirus (HPV) and cervical cancer, efforts to develop an effective prophylactic vaccine to prevent high-risk HPV infections have been at the forefront of modern medical research. HPV causes 530,000 cervical cancer cases worldwide, which is the second most common cause of cancer deaths in women; a worldwide collaboration among epidemiologists, molecular biologists, vaccinologists, virologists, and clinicians helped lead to the development of two highly effective prophylactive HPV vaccines. The first, Gardasil, is a quadrivalent vaccine made up of recombinant HPV L1 capsid proteins from the two high-risk HPV types (16/18) responsible for 70% of cervical cancer cases as well as two low-risk HPV types (6/11) which are the causative agent for genital warts. The second, Cervarix, is a bivalent vaccine that was FDA approved three years after Gardasil and is also composed of L1 capsid proteins from HPV types 16/18. This review article focuses on the safety and efficacy data of both FDA-approved vaccines, as well as highlighting a few advances in future HPV vaccines that show promise in becoming additional treatment options for this worldwide disease.

## 1. Introduction

One of the most groundbreaking scientific discoveries in the last 50 years is the causal association between human papillomavirus (HPV) and cervical cancer. Harald zur Hausen, who discovered HPV 16 in cervical cancer specimens, received a Nobel Prize in Physiology and Medicine in 2008 for this accomplishment. This discovery catapulted HPV into the forefront of medical research in an effort to develop an effective prophylactic vaccine to prevent HPV and thus HPV associated cancers [1].

HPV, which is a non-enveloped, circular, double stranded DNA virus, is part of the papovavirus family [2]. HPV infects the basal cells of the epithelial mucosa. There are over a 100 HPV genotypes, differentiated by varying viral genome sequences [3]. Clinically, these diverse genotypes can be differentiated into cutaneous and mucosal types. The cutaneous types are divided into those that occur in the general population (HPV 1, 2 and 4) and those that cause epidermodysplasia verruciformis (HPV 5 and 8). The mucosal types are divided into low, moderate and high risk types (risk determined by its carcinogenic potential). Low risk types are HPV 6 and 11, moderate risk types are HPV 31, 33, 35, 52, 58 and 67 and high risk types are 16 and 18 [4]. The most common HPV types found in women are 16, 18, 31, 52 and 58 [5].

Two HPV proteins, E6 and E7, are implicated in viral oncogenesis. Both proteins target tumor suppressor genes, specifically E6 targets TP53 and E7 targets retinoblastoma proteins. E6 promotes the degradation of TP53, which is essential in promoting apoptosis of cells with error-ridden DNA [6]. E7 promotes the degradation of retinoblastoma proteins, which leads to the expression of S-phase proteins. These proteins trigger cells to reenter the cell cycle and begin DNA synthesis [7].

HPV is the most common sexually transmitted infection [8]. The worldwide incidence of HPV is estimated to be 11%–12% in women without cervical irregularities. Higher incidences are found in sub-Saharan Africa, Eastern Europe and Latin America (Forman). The first peak of HPV is in patients aged 25 years and younger and in North America, South America and Africa a second peak exists in patients aged 45 years or older [5]. Risk factors include sexual intercourse at a young age, high number of sexual partners, low socioeconomic status, young age, multiparity, circumcision, condom use, oral contraceptive use, smoking, immune suppression, and genetic abnormalities in the human leukocyte antigen system.

Noncancerous cutaneous manifestations of HPV are the common warts (verruca vulgaris), plantar warts, plane warts, anogenital warts and epidermodysplasia verruciformis. Mucosal manifestations of HPV are oral warts, condylomata, focal epithelial hyperplasia (Heck’s disease), nasal and conjunctival papillomas, laryngeal papillomas and cervical lesions [4]. A causal link has been found between HPV and cervical, penile, vulvar, vaginal, anal and oropharyngeal carcinoma [9]. Specifically, HPV causes 530,000 cervical cancer cases worldwide yearly as well as 90% of anal cancers and approximately 50% of oropharyngeal, penile, vaginal and vulvar cancers. HPV 16 and 18 cause 70% of cervical cancer cases [10].

As detailed above, HPV imposes an immense clinical burden on the world’s population. Although Papaniclolau smears have reduced cervical cancer incidence by 80%, it is still the second most common cause of cancer deaths in women. A key factor in reducing the morbidity and mortality associated with HPV lies in prevention of initial HPV infection. This has led to a worldwide collaboration among epidemiologists, molecular biologists, vaccinologists, virologists and clinicians to develop a highly effective prophylactic HPV vaccine.

## 2. Quadrivalent Vaccine

### 2.1. Preparation

The quadrivalent HPV vaccine, Gardasil, is produced by Merck, Inc. (Whitehouse Station, NJ, USA). It contains recombinant HPV L1 capsid proteins from two high-risk HPV types, HPV-16/18, and two low-risk HPV types, HPV-6/11, and uses amorphous aluminum hydroxyphosphate sulfate as adjuvant [11].

Recombinant forms of L1 major capsid proteins have been shown to form HPV virus-like particles (VLPs) that are empty icosahedral shells identical in structure to native HPV virons [12]. These VLPs have been shown to confer HPV type-specific immunity without the delivery of potentially harmful oncogenic and structural production genes [13]. They may induce some degree of a cell-mediated host immune response. However, this cell-mediated response is considered irrelevant to cancer prevention as infected basal epithelial layers are known not to express significant levels of the L1 major capsid proteins until after the development of malignant lesions [14].

The L1 protein vaccine antigens are recombinant proteins expressed in the *Saccharomyces cerevisiae* yeast for the quadrivalent vaccine [13]. Based on the composition of the adjuvant in the quadrivalent vaccine, the placebos in the studies evaluating the safety and efficacy of quadrivalent HPV vaccines detailed below were saline based and aluminum hydroxyphosphate sulfate containing adjuvants.

### 2.2. Phase I Safety Data/Results

Initially, Merck conducted four Phase I studies (Supplementary Information Table S1)] in North America evaluating the safety and immune response of the individual HPV VLPS 11, 16, and 18 at varying doses [15]. These four studies including Study 001 which evaluated the monovalent HPV VLP serotype 11 in 140 subjects, Study 002 which evaluated serotype 16 in 109 subjects, Study 004 which evaluated serotype 16 in 480 subjects, and Study 006 which evaluated serotype 18 in 40 subjects. Studies 001 and 002 enrolled a total of 249 subjects with approximately 200 receiving the vaccine at varying doses, and the studies demonstrated a clear dose response. Phase I studies demonstrated a greater immune response in subjects receiving the 20 μg, 40 μg, or 50 μg dose than in those who received the 10 μg dose. However, there appeared to be a limit in the advantage of increasing the dose as there was no increase in immune response observed with the administration of an 80 μg dose compared to the 50 μg dose or the administration of a fourth dose.

### 2.3. Phase II

Study endpoints for Phase II (Supplementary Information Table S2) and III (Supplementary Information Table S3) studies of the quadrivalent vaccine included seroconversion, prevention of persistent HPV infection, prevention of CIN 1-3 development, prevention of vulvar and vaginal lesions, and prevention of genital warts [16,17,18,19,20,21]. Two phase II study protocols by Merck, Protocol 005 and 007, evaluated the efficacy of the monovalent HPV VLP, serotype 16, and quadrivalent vaccine, serotypes 6, 11, 16, and 18, respectively, in preventing the clinical endpoint of persistent HPV infection, defined by two or more consecutive cervicovaginal samples at visits 4 months apart positive for HPV by PCR.

Study Protocol 005 was an early Phase II, double-blind, placebo-controlled, randomized study conducted by Merck, evaluating the monovalent VLP serotype 16 [15]. It was conducted in 2409 subjects in North America. The purpose of the study was to further characterize differences in safety and the immune response to the monovalent HPV serotype 16 vaccine at varying doses.

Study Protocol 007 by Merck was a double-blind, randomized, placebo-controlled, Phase II study of the quadrivalent 6/11/16/18 vaccine of a total of 2409 subjects in North America, Latin American, and Europe evaluating the safety and efficacy of low, medium, and high dose quadrivalent vaccine groups *versus* placebo.

As part of this 007 protocol, Villa *et al.* published in 2005 a multi-center, double-blind, placebo-controlled study of 277 women randomized to the low-dose quadrivalent vaccine group (20/40/40/20 μg dose formulation) and 275 to the placebo group (either 225 μg or 450 μg of the aluminum hydroxyphosphate sulfate adjuvant) [22]. Follow-up included regular gynecological exams, cervicovaginal HPV DNA testing, serum HPV antibody testing, and Pap testing. Additionally, 241 subjects received an additional two years of follow-up for a total of five years of follow up [23].

#### 2.3.1. Safety

The quadrivalent vaccine was concluded to be safe compared to placebo based on the results of Protocol 005, Protocol 007, and the Protocol 007-based study by Villa *et al.* both at three year and five year follow up. Adverse events with an increased rate in those administered the vaccine *versus* placebo in Phase II trials included pain and erythema at the site of administration [24]. Protocol 007 did report an increase in the proportion of subjects with adverse events in the higher dose group compared to those administered lower doses, and Villa *et al.* found an increase of injection-site adverse events from 3% with placebo to 6% with vaccine. However, these studies reported no vaccine-related serious adverse events, and the quadrivalent vaccine at high, intermediate, or low doses was found to have an acceptable safety profile. No dose of vaccine was excluded from Phase III trials solely based on  safety profile [22].

#### 2.3.2. Efficacy

Study 005 found initial evidence of the efficacy of monovalent HPV 16 VLP vaccination in preventing HPV-16-associated disease. Of subjects with no evidence of HPV infection at baseline, none of the 753 subjects in the vaccine group developed HPV-16-associated CIN while 16 of the 750 subjects in the placebo group developed HPV-16-associated CIN.

A secondary analysis of Study Protocol 007 evaluating the secondary clinical endpoint of persistent HPV infection included only women who had no evidence of HPV infection with negative serologic testing at baseline [15]. This analysis found four out of 235 subjects in the low dose group, seven out of 232 subjects in the medium dose group, three out of 234 subjects in the high dose group, and 35 out of 233 subjects in the placebo group to have persistent HPV infection.

The results of the Protocol-007 based Villa *et al.* study concluded that the incidence of persistent of infection or disease with HPV types 6, 11, 16, or 18 fell by 90% (95% confidence interval (CI) = 70.7%–97.3%) in those who received low-dose vaccine (20/40/40/20 μg for HPV serotypes 6/11/16/18, respectively) compared to those who received placebo with follow-up for three years [22]. Of those with persistent infection in the vaccine group, three had HPV-16 detected at the last visit without further observed persistence, and one had persistent infection with HPV-18 detected at 12 and 18 months but not at 24, 30, or 36 months. The results of the five-year follow up study demonstrated a 96% reduction of HPV 6/11/16/18 persistent infection, 100% efficacy of HPV 6/11/16/18-related precancerous cervical dysplasia and genital warts (CI = 12%–100%), and titers that remained at or above those following natural infection at five years. The persistence of immunity at five years supported the utility of HPV vaccination of adolescents and young adults [23].

The low dose group—20/40/40/20 μg for HPV serotypes 6/11/16/18, respectively—was chosen to be studied further as part of clinical development in Phase III studies of the quadrivalent HPV vaccine due to the combination of the greater proportion of subjects in the higher dose groups with reported adverse events compared to lower dose groups and comparable efficacy of lower dose groups to higher dose groups.

### 2.4. Phase III

In Phase III trials, the intervention being investigated was a three-dose series of quadrivalent HPV vaccine administered at months 0, 2, and 6 *versus* placebo administered at months 0, 2 and 6 (Supplementary Information Table S3). The Future I and Future II trials were randomized, double-blind, placebo-controlled clinical trials of the effectiveness of the quadrivalent vaccine that enrolled 17,622 women without baseline HPV status consideration [25]. The Future I trial enrolled 5455 women aged 16–23 years from North American, Latin America, South America, Europe, Asia and Australia who had normal baseline pelvic examinations with no HPV prescreening before randomization, and the Future II trial enrolled 12,167 women aged 16–23 years from North America, South America, Europe, and Asia who had normal baseline pelvic examinations with no HPV prescreening before randomization [15]. The clinical endpoints investigated in both studies included CIN 2/3+ and genital lesions caused by HPV. Additionally, the Future I study sought to determine the efficacy of the quadrivalent vaccine against the incidence of HPV 6/11/16/18-associated CIN, adenocarcinoma *in situ*, and cervical cancer in women with no evidence of previous infection with HPV at baseline in order to provide evidence of potential utility for cervical cancer prevention in a population of adolescent and young adult females. The average follow-up time of patients in the Future I study was three years [16]. Eighty-three percent (*n =* 2261) of the women in the vaccine group and 83% (*n =* 2279) of the women in the placebo group were followed for vulvar, vaginal, or perianal disease, and 82%, (*n =* 2241) of the vaccine group and 83% (*n =* 2258) of the placebo group were followed for cervical disease.

Olsson *et al.* conducted a Phase III, double-blind, placebo-controlled study of 552 women aged 16–23 years that randomized 276 women to the three-dose regimen of quadrivalent HPV vaccine and the other 276 women to placebo. This study had an average of three years of follow-up of all study subjects and included an additional two years of follow-up, for a total of five years of follow-up, in 114 subjects in the vaccine group and 127 subjects in the placebo group [26]. This study’s objective was to determine the extent of immune memory after quadrivalent vaccine administration. The participants in the five-year follow-up group were challenged at month 60 with a dose of the quadrivalent HPV vaccine to assess their immune memory.

#### 2.4.1. Safety

Similar to Phase II studies, a higher rate of localized adverse events at the injection-site with the quadrivalent HPV vaccine *versus* placebo was noted in both the Future II study and Future I study (87% *versus* 77%) [25]. In all Phase III studies, systemic and serious adverse events were similar between vaccine and placebo groups [16,25]. In the Future II study, the adverse events with a reported increased proportion in the vaccination group compared to the placebo group included headache (11.1% *versus* 10.7%), pyrexia (5.5% *versus* 4.6), nasopharyngitis (2.6% *versus* 2.3%), and nausea (2.9% *versus* 2.5). In the Future I study, the most common inject-site reaction was pain at the injection-site with a 10% risk difference (95% CI = 7.8–12.1). There was also reportedly an increased rate of fever in the range of 100 °F (37.8 °C) and 102 °F (38.9 °C) in the vaccine group (13.3% *versus* 10.3%) with a risk difference of 3.0% (95% CI = 1.3–4.8).

#### 2.4.2. Efficacy

The Future II study concluded that the quadrivalent vaccine was 100% effective in preventing incident CIN 2/3 and cervical adenocarcinoma in situ caused by HPV-16 and HPV-18 in women negative for HPV at enrollment (95% CI = 79–100) and 94% effective in preventing vulvar or vaginal HPV-related lesions (95% CI = 81%–99%). This study demonstrated the protection that quadrivalent vaccination provides, in women positive for 1–3 of the HPV vaccine types, against neoplasia and genital lesions caused by the remaining HPV types, supporting the utility of quadrivalent HPV vaccination in the general population without prescreening for HPV infection.

The Future I study demonstrated 100% efficacy for all co-primary endpoints including the prevention of precancerous and cancerous lesions of the cervix, vagina, and vulva and of genital warts associated with HPV 6/11/16/18 following the administration of the three-dose quadrivalent vaccine regimen with an average follow-up time of two years [27]. This study also performed an intention-to-treat analysis of infection or disease caused by both vaccine and non-vaccine type HPV. This analysis showed a 34% rate of reduction of any vulvar, vaginal, or perianal lesions regardless of the causal HPV type (95% CI = 15–49), and a 20% rate of reduction of cervical lesions regardless of the cervical HPV type (95% CI = 8–31) [16]. Thus, the Future I study concluded that the quadrivalent vaccine had a significant effect on the prevention of HPV-associated anogenital disease in young women, with a greater effect on vaccine-type HPV-associated lesions and a lesser but still significant reduction of HPV-associated lesions regardless of HPV type.

The Olsson *et al.* [26] study concluded that although serum anti-HPV levels declined post-vaccination and plateaued at month 24 with a stable level observed at month 60, anti-HPV levels rose dramatically following the administration of a challenge dose of vaccine at month 60. The one-week post-challenge levels reached levels observed 1 month after the completion of the three-dose vaccine series, and one-month post-challenge levels were higher than the levels at one month following three-dose vaccination series completion. Thus, this study suggests that immune memory is present, and the vaccine is efficacious for at least five years.

### 2.5. FDA Approval

In June 2006, the quadrivalent vaccine received FDA approval for use in girls and women aged 9–26 years for the prevention of CIN 1–3, cervical adenocarcinoma *in situ*, vulvar intraepithelial neoplasia grades 2 and 3, vaginal intraepithelial neoplasia grades 2 and 3, genital warts caused by HPV-6 and HPV-11, and cervical, vulvar, and vaginal cancers caused by HPV-16 and HPV-18 [28]. In October 2009, the FDA expanded the indication for use of the quadrivalent HPV vaccine to boys and men aged 9–26 years to protect against HPV infection and genital warts caused by HPV-6 and HPV-11 [29].

### 2.6. Further Data from Clinical Trials

More recent studies have investigated the efficacy of the quadrivalent vaccine in a younger population of adolescent girls aged 10–15 years (Supplementary Information Table S4). There is level 3 evidence, based on four studies, that supports the claim that the three-dose quadrivalent vaccine decreases vaccine type HPV-related infection and disease in young adolescent girls. Brotherton *et al.* conducted a study in Australia that compared the incidence of CIN2+ and adenocarcinoma in situ in women 12–26 years at two time points, three years prior to the implantation of free quadrivalent HPV vaccination in women aged 12–26 years in 2007 in Australia and two years after the implementation of that program [30]. This study found a 0.38% decrease in the incidence of CIN2+ and adenocarcinoma *in situ* in girls 12–18 years two years after program implementation compared to three years prior to implementation, and no significant difference in those 18–26 years of age. The study concluded that the quadrivalent vaccine program lead to a reduced incidence of high-grade cervical lesions in adolescent girls less than 18 years of age.

Crowe *et al.* carried out a case-control study without clinical outcomes of 108,353 women aged 12–26 years in Australia based on the results of their first Pap smear with 1062 cases of CIN2+ or adenocarcinoma *in situ*, 10,887 cases of another cervical abnormality noted either on cytology or histology, and 96,404 controls with no evidence of cervical lesions [31]. The study results concluded that completion of the three-dose quadrivalent vaccine, when compared to no vaccine, was associated with a decreased risk of CIN2+ or adenocarcinoma in situ (adjusted odds ratio (OR) = 0.54, 95% CI = 0.43–0.67) with vaccine efficacy of 46% and number needed to vaccinate of 125. A decreased risk of other cervical abnormalities (adjusted OR = 0.66, 95% CI = 0.62–0.7) was also seen, with vaccine efficacy of 34% and number need to vaccinate of 22.

Kahn *et al.* performed a before and after surveillance study with no clinical outcomes of a group of 13–26 year old females known to be sexually active, 368 of whom were in the prevaccination group without HPV vaccine, and were compared to 409 patients in the post vaccination group [32]. The prevalence of vaccine-type HPV (HPV serotypes 6, 11, 16, and 18) were assessed in both groups and was found to be 31.7% *versus* 13.4% in the post vaccination group (*p <* 0.0001). The study concluded that quadrivalent HPV availability in Australia was associated with a decreased prevalence of vaccine-type HPV in 13–26 years old girls and women.

Finally, Herweijer *et al.* performed a retrospective cohort of 1,045,165 females aged 10–24 years from the general population of Sweden with a mean follow-up of 3.8 years [33]. This study found that vaccination with three doses of the quadrivalent vaccine when the first dose was between age 10 and 19 years was associated with a decreased risk of condyloma when compared to other groups including no vaccine (incidence relative risk (RR) = 0.2, 95% CI = 0.17–0.23), one dose (incidence RR = 0.37, 95% CI = 0.28–0.48), and 2 doses (incidence RR = 0.63, 95% CI = 0.48–0.82).

In men, a randomized control trial of 4065 men aged 16–26 years randomized to either quadrivalent HPV vaccine or placebo that was administered at day 1, month 2, and month 6, with a median follow-up of 2.9 years [34]. The results of the study included a 97.5% seroconversion rate within one month of the third vaccine dose. The rates of external genital lesions for the quadrivalent vaccine group *versus* placebo group were 0.8 *versus* 2 for external genital lesions (*p <* 0.05), 0.45 *versus* 1.11 for HPV-6 associated external genital lesions (*p <* 0.05), 0.13 *versus* 0.52 for HPV-11 associated external genital lesions (*p <* 0.05), 0.52 *versus* 1.58 for HPV-6 or HPV-11 associated condylomata acuminata (*p <* 0.05), and 3.61 *versus* 6.92 for HPV-6, 11, 16, or 18 persistent infection (*p <* 0.05). There was no statistically significant reduction in HPV-16 or HPV-18 associated external genital lesions or perineal intraepithelial neoplasia lesions.

Palefsky *et al.* performed a randomized trial of 602 healthy men who have sex with men aged 16–26 years who were either randomized to the quadrivalent HPV vaccine or to placebo [35]. The efficacy of the quadrivalent HPV vaccine was 50.3% against anal intraepithelial neoplasia (AIN) associated with HPV infection of any type, 25.7% against vaccine-type HPV-associated AIN, 54.2% against grade 2+ vaccine-type HPV-associated AIN, and 59.4% against persistent HPV-6, 11, 16, or 18 anal infection. The study concluded that the quadrivalent vaccine might be effective in producing immunity to prevent AIN in men who have sex with men aged 16–26 years.

## 3. Bivalent Vaccine

### 3.1. Preparation

A bivalent HPV vaccine, Cervarix, is produced by GlaxoSmithKline (Rixensart, Belgium). It contains recombinant HPV L1 capsid proteins from the two high-risk HPV types, HPV-16/18 [36]. L1 capsid proteins are made using a baculovirus-insect cell expression system. The same principles of VLP formation with type-specific host immunity development discussed with the quadrivalent vaccine also apply to the L1 capsid protein of the bivalent vaccine. In addition to the bivalent rather than quadrivalent composition of the vaccine, the other difference in the bivalent vaccine compared to the quadrivalent vaccine is the adjuvant used. The bivalent vaccine uses the proprietary adjuvant, aluminum hydroxide with 3-deacylated monophosphoryl lipid A (ASO4) [24]. ASO4 in the bivalent vaccine is an aluminum-salt that also acts as a Toll-like receptor (TLR) 4 agonist and reportedly produces a superior antibody response compared to the traditional aluminum hydroxyl-phosphate sulfate adjuvant in the quadrivalent vaccine [14]. For studies of the safety and efficacy of the bivalent vaccine the Hepatitis A vaccine was used as placebo.

### 3.2. Phase I Safety Data/Results

HPV-002 was a Phase I, randomized, open-label study of 49 women aged 18–30 in the US to assess the safety and immunogenicity of the monovalent HPV-16 and HPV-18 and bivalent HPV-16/18 VLP vaccines formulated with AS04 as the adjuvant. Of the 49 subjects, 12 received monovalent HPV-16 (20 μg), 12 received monovalent HPV-18 (20 μg), and 25 received bivalent HPV-16/18 (20/20 μg) VLP vaccines [37]. Doses were administered at 0 and 28 days for all subjects with analysis at 56 days and an additional dose at 112 days with an analysis at 140 days for eight subjects in the monovalent HPV-16 VLP group. There were no limiting toxicities or safety concerns with the HPV-16, HPV-18, or HPV-16/18 VLP vaccines noted in this study (Supplementary Information Table S5).

Antibody and cell-mediated immune responses were noted with the HPV-16, HPV-18, and HPV-16/18 VLP vaccines after the administration of the second dose. Of note, in the bivalent vaccine, there was efficacy of the HPV-16 and HPV-18 VLPs in generating an immune response comparable to the response generated by the individual vaccine components in the monovalent vaccines. The third dose given to the eight monovalent HPV-16 vaccine recipients led to a further increase in anti-HPV antibody levels in all cases, suggesting the utility of a third dose. A follow-up study through 4.5 years after administration of the first dose found anti-HPV-16 antibody levels to be detectable in 33% of subjects and the persistence of specific Interferon-gamma responses for a range of 2–4.5 years in all subjects.

### 3.3. Phase II

The measures of outcome of Phase II and III trials of the bivalent vaccine are similar to those discussed for the quadrivalent vaccine but exclude the prevention of genital warts [24]. Two other studies included Phase I but also Phase II trails, HPV-003 and HPV-004 (Supplementary Information Table S5). The HPV-003 Study Group was a Phase I/II double-blind, randomized trial of 61 women aged 18–30 years to assess the safety and immunogenicity of the bivalent HPV-16/18 (20/20 μg) VLP vaccine [37]. Subjects were randomized to either the bivalent vaccine or to aluminum hydroxide control, which were administered at day 0, 30, and 180 days, and patients were followed for one year.

HPV-004 was a Phase II double-blind, randomized trial with 60 female subjects aged 18–30 years in the United Stated. The purpose of this study was to compare the safety and immunogenicity of the bivalent vaccine with two different adjuvants, AS04 and aluminum hydroxide, as well as without adjuvants. The 60 healthy female subjects were randomized to either bivalent vaccine with AS04, bivalent vaccine with aluminum hydroxide, or bivalent vaccine without adjuvant, and vaccine administration was at 0, 30, and 180 days. The patients were followed for two years.

The HPV-005 study was a Phase II, double-blind, randomized trial of 209 women aged 18–30 years with no evidence of HPV-16 or HPV-18 infection at baseline. The HPV-005 was a dose-range study to characterize the safety efficacy of the bivalent vaccine at different doses. The subjects were randomized to a 2:2:2:1 ratio to either receive 12 μg with AS04, 40 μg with AS04, 120 μg with AS04, or 40 μg with aluminum hydroxide on days 0, 30, and 180.

The HPV-007 Study Group was a double-blind, placebo-controlled trial with subjects including HPV-16/18-negative women aged 15–25 years with normal cervical cytology with 560 randomized to the vaccine group and 553 randomized to the placebo group [38]. Additionally, 393 of the vaccine group and 383 of the placebo group were included in the follow-up study.

#### 3.3.1. Safety

The safety profiles of the bivalent vaccine and placebo group were found to be similar, and there were no reported vaccine-related serious adverse events [37,38]. The HPV-004 study did not find significant differences in the proportions of study subjects in the AS04, aluminum hydroxide, or no adjuvant groups with serious or systemic adverse events. A higher rate of local injection-site reactions such as pain was reported with the group with AS04 adjuvant. The HPV-005 study found the 12 μg with AS04, 40 μg with AS04, 120 μg with AS04, and 40 μg with aluminum hydroxide formulation to all be generally tolerated. The only adverse event that was found to be proportional to increasing vaccine doses was local injection-site reaction.

#### 3.3.2. Efficacy

The HPV-003, HPV-004, and HPV-005 studies had similar immunogenicity results to Phase I studies, with an immune response demonstrated to both HPV-16 and HPV-18 after the second dose of bivalent vaccine, with antibody levels rising after the administration of a third dose [37]. A study specific finding of the HPV-003 study was that in women with evidence of prior HPV-16 and/or HPV-18 infection, the bivalent vaccine did not increase the clearance rate of HPV-16 or HPV-18 viral DNA compared to placebo. The HPV-004 study determined that the AS04 adjuvant group had higher ELISA titers than the aluminum hydroxide or no adjuvant groups at day 210. This study also found that the kinetic profile of the antibodies generated against HPV-16 and HPV-18 following bivalent vaccine administration was comparable to the neutralizing antibody profile, with the greatest HPV-16 humoral response noted in the AS04 group. The HPV-005 study did not find a significant effect of VLP dose of the AS04 formulations on the cellular immune response; however, the ELISA titers at the three VLP doses formulated with AS04 suggested that the lowest dose (12 μg) may produce less of a humoral immune response. This was used by the sponsor to support the 40 μg dose of the bivalent vaccine with AS04 as the formulation chosen for further clinical development.

The HPV-007 study results included that vaccine efficacy against the incidence of HPV-16/18 infection was 93.3% (95% CI = 87.4–98.7), against 12-month persistent infection was 100% (95% CI = 81.8–100), and against CIN 2+ associated with HPV-16/18 was 100% (95% CI = 51.3–100). The efficacy against lesions independent of HPV DNA type was 71.9% (95% CI = 20.6–91.9). The study also found that anti-HPV-16 and anti-HPV-18 antibody concentration remained 12-fold or more times greater than that achieved after natural infection.

### 3.4. Phase III

The Phase III randomized, double-blind, controlled trial called PApilloma TRIal against Cancer In Young Adults (PATRICIA) (Supplementary Information Table S6) included a cohort of women aged 15–25 years regardless of baseline HPV status and sexual activity with 9319 randomized to the vaccine group and 9325 to the control group. The Total Vaccine Group-naïve group represented women before sexual debut with no baseline evidence of HPV infection with 5822 in the vaccine group and 5819 in the control group. Mean follow-up was 34.9 months after the third dose.

A masked, community-based study in two provinces of Costa Rica studied the safety and efficacy of the bivalent vaccine in 2189 women aged 18–25 years with preexisting HPV infection (HPV DNA-positive at enrollment) with 1088 women randomized to the vaccine group and 1101 to the placebo group. They were followed for six months [39].

A separate study performed in multiple centers across Europe and Russia studied the safety and efficacy in the form of immunogenicity of the bivalent vaccine in 773 females aged 10–14 years and 15–25 years to compare responses between the two age cohorts. Vaccine was administered according to previous dosing schedules and subjects were followed for seven months [40].

#### 3.4.1. Safety

PATRICIA found that the rates of serious adverse events, medical conditions, and new-onset chronic and autoimmune disease were similar between the bivalent vaccine and control groups [18].

#### 3.4.2. Efficacy

PATRICIA determined that the vaccine efficacy against HPV-16/18 associated CIN2+ was 92.9% (96.1% CI = 79.9–98.3) in primary analysis and 98.1 (96.1% CI = 88.4–100) in an analysis adjusting for probable causality in lesions with multiple oncogenic types [18]. This study found the efficacy against CIN2+, irrespective of HPV DNA type in the lesions, to be 30.4% (96.1% CI = 16.4–42.1) in the total vaccine group and 70.2% (96.1 CI = 54.7–80.9) in the Total Vaccine Group-naïve group. The efficacy against CIN2+ associated with 12 non-vaccine oncogenic HPV types was 54.0% (96.1 CI = 34.0–68.4). The bivalent vaccine shows efficacy against both HPV-16/18 associated CIN2+ as was expected, but also demonstrated efficacy in preventing CIN2+ associated with non-vaccine oncogenic HPV types including HPV-31, HPV-33, and HPV-45, suggesting cross-protecting to other oncogenic HPV types. The bivalent vaccine showed greater cross-reactivity with other oncogenic HPV types compared to the quadrivalent vaccine [16,17,18].

The study of 2189 HPV-positive Costa Rican women showed no evidence of accelerated viral clearance at six or 12 months (Supplementary Information Table S6). The viral clearance at six months was 33.4% in the vaccine group and 31.6% in the control with vaccine efficacy of 2.5% (95% CI = −9.8–13.5). At 12 months, the clearance was 48.8% in the vaccine group and 49.8% in the control group with vaccine efficacy of −2.09% (95% CI = −24.3–16.3). Thus, this study concluded that the administration of the bivalent vaccine does not accelerate clearance and should not be used for the indication of treating pre-existing infection.

The study comparing safety and immunogenicity of ASO4 between females aged 10–14 and 15–25 years showed a similar safety profile as that of the PATRICIA study and indistinguishable profiles between the two age cohorts. Seroconversion found to be similar between the two age cohorts. Mean antibody titers were approximately twice as high in females aged 10–14 compared to females 15–25, suggesting longer antibody persistence and supporting prophylactic vaccination of younger adolescent females [40] (Supplementary Information Table S6).

### 3.5. FDA Approval

Despite approval in 77 other nations since 2007, the bivalent HPV vaccine did not receive FDA approval until October 2009. It is now approved in girls and women aged 10–25 years for the prevention of cervical cancer, CIN1, CIN2/3, and cervical adenocarcinoma in situ caused by HPV types 16 and 18 [36,41]. There is no FDA approval for use in boys and men.

### 3.6. Further Data from Clinical Trials

Follow-up to Harper’s trial showed that the bivalent vaccine is safe, immunogenic, and provides protection against HPV-16/18 infection and associated cervical lesion for up to 4.5 years [20] and 6.4 years [21].

Post-licensure safety data of the bivalent HPV vaccine since licensure has been comparable to pre-licensure reports in terms of the safety profile and reported adverse events following administration with no evidence to support any immune-mediated disease post-vaccination [42]. The incidence of immune mediated disease such as Bell’s palsy and confirmed Guillain-Barré syndrome were comparable to those expected in the general population. The post-licensure surveillance data still support the conclusion of the pre-licensure data that the bivalent HPV vaccine is appropriate for use in adolescent girls and women for the prevention of HPV-related infection and disease based on its benefit-risk profile.

There have been no studies of the efficacy of the bivalent vaccine in men to date, or efficacy in the prevention of head and neck squamous cell carcinoma in men or women.

### 3.7. Comparison with Quadrivalent Vaccine

Due to the similarities between the quadrivalent and bivalent vaccines, individuals seeking prevention from HPV-related diseases may have difficulty choosing which vaccine to receive. Both vaccines are efficacious in establishing protection, and the choice of vaccine is typically up to the discretion of healthcare providers. However, differences in immunogenicity exist between the vaccines. While unable to prevent genital warts caused by HPV types 6 and 11, the bivalent vaccine was shown in an observer-blind study to induce higher mean antibody titers compared to the quadrivalent vaccine one month after completion of vaccination course [43]. Further long-term studies are needed to determine if this difference in immunogenicity is a serologic correlate for longer efficacy post-vaccination.

## 4. Investigational Vaccines

While the L1 peptide vaccines Gardasil and Cervarix have been instrumental in decreasing rates of HPV infection worldwide, important drawbacks exist with both vaccines. Protection against non HPV-16 and HPV-18 oncogene types has not been clearly established, leaving room for other high-risk mucosal HPV infections. Additionally, the cost of preparation of both vaccines creates an obstacle for instituting vaccination programs in developing countries where HPV is prevalent [9,44]. Thus, several investigational vaccines that circumvent these drawbacks have been developed and studied in animal or clinical trials.

### 4.1. Multivalent Vaccine

A nine-valent vaccine (Gardasil 9) was developed by Merck, Inc (Kenilworth, United States) to address the lack of protection from other oncogene types not covered by Gardasil and Cervarix. In addition to HPV types 6, 11, 16, and 18, Gardasil 9 covers HPV types 31, 33, 45, 52, and 58. An international randomized double-blinded phase III trial initiated in 2007 comparing this nine-valent vaccine to Gardasil successfully enrolled 14,204 female subjects ages 16–26. Primary outcomes of incidence of HPV-type disease, mean antibody titers, and safety profiles were evaluated for subjects receiving Gardasil 9 and Gardasil. Gardasil 9 was shown to be 97% effective in the prevention of cervical, vaginal, and vulvar neoplasms caused by the five additional HPV types, as well as having similar effectiveness to Gardasil in the prevention of diseases caused by HPV types 6, 11, 16, and 18. Mean antibody titers to HPV types 6, 11, 16, and 18 were similar between Gardasil 9 and Gardasil. Similar safety profiles were demonstrated between the two vaccines in the trial, with injection site reaction being the most common adverse event. Separate studies evaluating Gardasil 9 in females and males ages 9–15 demonstrated similar mean antibody titers compared to that seen in subjects ages 16–26. These promising results led the FDA in December 2014 to approve Gardasil 9 for the prevention of cervical, vaginal, vulvar, and anal cancers caused by HPV types 16, 18, 31, 33, 45, 52, and 58 and for the prevention of genital warts caused by HPV types 6 and 11 for females ages 9–26 and males ages 9–15 [45],

### 4.2. L2 Vaccine

A vaccine against the HPV minor capsid protein L2 has been recently suggested as an alternative or concomitant prophylaxis against HPV infection. The L2 protein plays fundamental roles in HPV infection, including immune escape, viral genome packaging, and viral entry into host cells [46]. An advantage of vaccination against L2 is the potential for broad-spectrum protection due to the homologous structure of the protein that is shared across numerous PV types [47]. Several animal [48,49,50] and human [51,52,53] trials have been performed which demonstrated immune responses across several papillomavirus oncogenic types with a single vaccine. In particular, Gambhira showed that patients who received an investigational HPV fusion vaccine including L2 developed dose-dependent antibodies to HPV types 16 and 18 of different phylogenetic species each with unique biological properties, suggesting broad-spectrum protection. While clinical response was limited due to low antibody titers, evidence of HPV protection in animal models have sparked interest in developing an adjuvant L2 vaccine to boost seroconversion. Additionally, high numbers of the L2 vaccine can be produced in bacteria or by synthetic methods [54,55]. This potential for a low-cost, broad-spectrum vaccine has continued to support the utilization of L2 protein for HPV vaccination.

### 4.3. Therapeutic HPV Vaccine

Medical uses for vaccines have grown beyond prophylaxis with the advent of therapeutic vaccines targeting diseases that have been already acquired. Since the turn of the century there have been multiple studies involving therapeutic tumor vaccines targeting breast, lung, pancreatic, colorectal, and blood cancers [56]. Therapeutic DNA vaccines against HPV have recently been developed and tested in several clinical trials. These vaccines target the E6 and E7 viral peptides, which are fundamental proteins for tumorigenesis and tumor maintenance within infected epithelial cells; immune responses against these viral peptides have been shown to control CIN and other HPV-related diseases [57]. Whereas Gardasil and Cervarix induce prophylactic immunity via the production of antibodies, therapeutic HPV vaccines trigger antigen-specific action by cytotoxic T lymphocytes (CTLs). These CTLs recognize and attack epithelial cells infected by HPV as well as tumor cells. Additionally, DNA vectors themselves have been demonstrated to trigger innate inflammatory responses due to an immunostimulatory effect [58]. Compared to peptide and subunit vaccines, DNA vaccines are more stable at wider temperature ranges, which inherently lowers production and distribution costs. These attributes have made E6/E7 DNA vaccines attractive investigational options for treating existing HPV infections. A 2010 study using a synthetic E6/E7 peptide vaccine involving patients with PV16-associated grade 3 vulvar intraepithelial neoplasia showed complete regression in 50% of vaccinated subjects compared to 1.5% spontaneous regression rates [59] in lesions less than 9.5 cm^2^. The study also reported continued HPV16-specific T-cell memory through a two-year follow-up period. A separate phase I study in 2012 using the experimental vaccine VGX-3100 (SynCon^®^, Inovio, Plymouth Meeting, PA, United States) given to individuals with history of grade 2–3 cervical intraepithelial neoplasia showed that T-cell immunogenicity of a E6/E7 vaccine could be vastly increased with delivery via electroporation [60]. Phase I results also showed an excellent safety profile and efficacy of induced HPV-specific T-cells in killing assays. A subsequent phase II trial with VGX-3100 involving patients with CIN 2–3 was recently completed in 2014 and showed positive efficacy data in causing regression of CIN and clearance of HPV infection; phase III testing has been planned for 2016. It is conceivable that an HPV therapeutic DNA vaccine as a new treatment option will exist in the near future for individuals with CIN and cervical cancer, either as solo therapy or in conjunction with current therapies [61,62,63].

## 5. Conclusions

While complete prevention against HPV infection remains to be achieved, important breakthroughs in the 21st century have brought modern medicine closer to the goal. The FDA-approved vaccines Gardasil, Cervarix, and Gardasil 9 have shown incredible efficacy in past and recent trials in prevention of neoplastic and pre-neoplastic lesions associated with HPV infection and will likely continue to play a large role in HPV prevention among young females and males. With the recent promising results of therapeutic HPV vaccines, treatments for those with existing HPV infections may be on the horizon. Together, these therapeutic options for patients will undoubtedly prove to be integral for global control of this important disease.

## Supplementary Files

Supplementary File 1
