# Supplementary material for: Safety and Efficacy Data on Vaccines and Immunization to Human Papillomavirus
Source: J Clin Med. 2015 Apr 3;4(4):614–33. doi: 10.3390/jcm4040614 (PMC4470159; doi:10.3390/jcm4040614)
Supplement: Supplementary File 1 [file jcm-04-00614-s001.pdf]

Supplementary Information

Table S1. Phase I Quadrivalent HPV Vaccine Trials.

Name	Subject Characteristics	Follow up Duration	Results
Study 001 [15]	N = 140 (Monovalent HPV-11 Vaccine and Placebo) Sex: Female	All Subjects: Dose 1: month 0, Dose 2: month 2, Dose 3: month 6	Safety: No limiting toxicities or safety concerns<br>Immunogenicity:<br>Greater immune response with higher monovalent HPV vaccine doses of 20 µg, 40 µg, or 50 µg compared to 10 µg dose<br>No increase in immune response with 80 µg dose compared to 50 µg dose
Study 002 [15]	N = 109 (Monovalent HPV-16 Vaccine and Placebo) Sex: Female	All Subjects: Dose 1: month 0, Dose 2: month 2, Dose 3: month 6	Safety: No limiting toxicities or safety concerns<br>Immunogenicity:<br>Greater immune response with higher monovalent HPV vaccine doses of 20 µg, 40 µg, or 50 µg compared to 10 µg dose<br>No increase in immune response with 80 µg dose compared to 50 µg dose
Study 004 [15]	N = 480 (Monovalent HPV-16 Vaccine and Placebo) Sex: Female	All Subjects: Dose 1: month 0, Dose 2: month 2, Dose 3: month 6	Safety: No limiting toxicities or safety concerns<br>Immunogenicity:<br>Greater immune response with higher monovalent HPV vaccine doses of 20 µg, 40 µg, or 50 µg compared to 10 µg dose<br>No increase in immune response with 80 µg dose compared to 50 µg dose
Study 006 [15]	N = 40 (Monovalent HPV-18 Vaccine and Placebo) Sex: Female	All Subjects: Dose 1: month 0, Dose 2: month 2, Dose 3: month 6	Safety: No limiting toxicities or safety concerns<br>Immunogenicity:<br>Greater immune response with higher monovalent HPV vaccine doses of 20 µg, 40 µg, or 50 µg compared to 10 µg dose<br>No increase in immune response with 80 µg dose compared to 50 µg dose

HPV: human papillomavirus; *N*: number of subjects.

Table S2. Phase II Quadrivalent HPV Vaccine Trials.

Name	Subject Characteristics	Follow up Duration	Results
Study 005 [15]	*N* = 2409 (1:1 Ratio of subjects to Monovalent HPV-16 to Placebo Groups)<br>Age range: 18–25 years<br>Sex: Female<br>Other Inclusion Criteria: 4 or fewer sexual partners	All Subjects:<br>Dose 1: month 0,<br>Dose 2: month 1,<br>Dose 3: month 6,<br>Follow up: 7 months	Safety:<br>No vaccine-related serious adverse events<br>Increased injection-site reactions compared to placebo<br>Efficacy: Of subjects with no evidence of HPV infection at baseline, 0/753 subjects in the monovalent vaccine group developed HPV-16-associated CIN compared to 16/750 subjects in the placebo group
Study 007 [22,23]	*N* = 2409 (1:1:1:1 Ratio of subjects to Low-dose, Medium-dose, High-dose, and Placebo Groups)<br>Age range: 16–23 years<br>Sex: Female<br>Other Inclusion Criteria: No previous abnormal pap smears, 4 or fewer male sex partners	All Subjects:<br>Dose 1: month 0,<br>Dose 2: month 2,<br>Dose 3: month 6,<br>Follow up: 3 years<br>241 subjects were followed an additional 2 years for 5-year total follow up	Safety:<br>No vaccine-related serious adverse events<br>Increased injection-site reactions compared to placebo<br>Increase in rate of adverse events in higher dose groups compared to lower doses<br>Acceptable safety profile at all doses<br>Quadrivalent vaccine found safe at both 3-year and 5-year follow up<br>Immunogenicity: at five-year follow up titers remained at or above the level following natural infection at 5 years<br>Efficacy:<br>Against the incidence of persistent HPV-6/11/16/18 infection at 3 years: 90% (95% CI = 70.7–97.3) with low-dose quadrivalent vaccine<br>Against the incidence of HPV-6/11/16/18-associated precancerous cervical dysplasia and genital warts at 5 years: 100% (95% CI = 12%–100%) with low-dose quadrivalent vaccine

HPV: human papillomavirus; *N*: number of subjects; CI: confidence interval; CIN: cervical intraepithelial neoplasia.

Table S3. Phase III Quadrivalent HPV Vaccine Trials.

Name	Subject Characteristics	Follow up Duration	Results
Future I [15,16, 22,27]	*N* = 5455 (Quadrivalent HPV Vaccine = 2721, Placebo = 2744)<br>Age range: 16–23 years<br>Sex: Female	All Subjects: Dose 1: month 0, Dose 2: month 2,<br>Dose 3: month 6,<br>Mean follow up: 3 years	Safety:<br>Similar rates of systemic and serious adverse events between vaccine and placebo groups<br>Higher rate of injection-site reactions with vaccine *versus* placebo<br>Increased rate of adverse events with vaccine *versus* placebo included injection-site pain with a 10% risk difference (95% CI =7.8–12.1) and fever in the range of 100°F and 102°F with risk difference of 3.0% (95% CI = 1.3–4.8)<br>Efficacy:<br>100% efficacy for prevention of HPV-6/11/16/18-associated precancerous and cancerous lesions of the cervix, vagina and vulva and genital warts<br>34% rate of reduction of any vulvar, vaginal, or perianal lesions regardless of causal HPV type (95% CI = 15–49)<br>20% rate of reduction of cervical lesions regardless of the cervical HPV type (95% CI = 8–31)
Future II [15,16]	*N* = 12,167 (1:1 Ratio of subjects in Quadrivalent HPV Vaccine and Placebo groups)<br>Age range: 16–23 years<br>Sex: Female	All Subjects: Dose 1: month 0, Dose 2: month 2, Dose 3: month 6,<br>Mean follow up: 1.4 years	Safety:<br>Similar rates of systemic and serious adverse events between vaccine and placebo groups<br>Higher rate of injection-site reactions with vaccine *versus* placebo<br>Increased rate of adverse events with vaccine *versus* placebo included headache (11.1% *versus* 10.7%), pyrexia (5.5% *versus* 4.6%), nasopharyngitis (2.6% *versus* 2.3%), and nausea (2.9% *versus* 2.5%)<br>Efficacy:<br>In women HPV-negative at baseline, 100% efficacy for prevention of incident HPV-16/18-associated CIN 2/3 and cervical adenocarcinoma in situ (95% CI = 79–100)<br>In women HPV-negative at baseline, 94% efficacy for prevention of vulvar or vaginal HPV-related lesions (95% CI = 81%–99%)

Table S3. *Cont.*

Olsson *et al.* [26]	N = 552 (Quadrivalent HPV Vaccine = 276, Placebo = 276) Age range: 16–23 years	All Subjects: Dose 1: month 0, Dose 2: month 2, Dose 3: month 6, Mean follow up: 3 years 114 subjects in quadrivalent vaccine group and 127 in placebo group were followed for 5 years and were challenged at month 60 with a 4th dose of quadrivalent HPV vaccine	Safety: Similar rates of systemic and serious adverse events between vaccine and placebo groups Immunogenicity: Anti-HPV levels decline post-vaccination and plateau at month 24 with a stable level at month 60 Dramatic rise of anti-HPV levels following the administration of a challenge dose at month 60 1-week post-challenge levels comparable to levels at 1 month following completion of the 3-dose vaccination series 1-month post-challenge levels higher than levels at 1 month following completion of 3-dose vaccination series

HPV: human papillomavirus; *N*: number of subjects; CI: confidence interval; CIN: cervical intraepithelial neoplasia.

Table S4. Post-Licensure Quadrivalent HPV Vaccine Studies.

Name	Subject Characteristics	Follow up Duration	Results
Brotherton *et al*. [30]	Data from Victoria Cervical Cytology Registry between 2003–2009 used to compare the incidence of CIN2+ and adenocarcinoma in situ before and after implementation of free quadrivalent HPV vaccination in women aged 12–26 years<br>Age range: 12–26 years<br>Sex: Female	Time point 1:3 years prior program implementation, Program implementation: 2007, Time point 2:2 years following program implementation	Efficacy:<br>0.38% decrease in the incidence of CIN2+ and adenocarcinoma in situ in girls aged 12–18 years when comparing 2 years after to 3 years prior to program implementation<br>No significant difference in girls aged 13–26 years
Crowe *et al*. [31]	Case-control of 108,353 Australians based on their first Pap smear (CIN2+ or adenocarcinoma *in situ* = 1062, another cervical abnormality on cytology or histology = 10,877, controls with no evidence of cervical lesions = 96,404)<br>Age range: 12–26 years<br>Sex: Female		Efficacy:<br>Decreased risk of CIN2+ or adenocarcinoma in situ when compared to no vaccine (adjusted OR= 0.54, 95% CI = 0.43–0.67) with vaccine efficacy of 46% and number needed to vaccinate of 125<br>Decreased risk of other cervical abnormalities when compared to no vaccine (adjusted OR= 0.66, 95% CI = 0.62–0.7) with vaccine efficacy of 34% and number needed to vaccinate of 22
Kahn *et al*. [32]	Surveillance study (Pre-vaccination group = 368, post-vaccination group = 409)<br>Age range: 13–26 years<br>Sex: Female		Prevalence: HPV-6/11/16/18 prevalence in the pre-vaccination group was 31.7% *versus* 13.4% in the post-vaccination group (p < 0.0001)

Table S4. *Cont.*

Herweijer *et al.* [33]	Retrospective cohort<br>*N* = 1,045,165<br>Age range: 10–24 years<br>Sex: Female	Mean follow up: 3.8 years	Efficacy:<br>Three-dose quadrivalent HPV vaccination with first dose between age 10–19 years associated with decreased risk of condyloma when compared to no vaccine (RR = 0.2, 95% CI = 0.17–0.23), one dose (RR = 0.37, 95% CI = 0.28–0.48), and 2 doses (RR = 0.63, 95% CI = 0.48–0.82)
Giuliano *et al.* [34]	Randomized control trial<br>*N* = 4065 (1:1 Ratio of subjects to Quadrivalent HPV Vaccine and Placebo Groups)<br>Age range: 16–26 years<br>Sex: Male	All Subjects: Dose 1: month 0, Dose 2: month 2, Dose 3: month 6, Mean follow up: 2.9 years	Immunogenicity: 97.5% seroconversion rate one month following vaccine series completion<br>Efficacy:<br>External genital lesion rates for vaccine group *versus* placebo group included 0.8 *versus* 2 for external genital lesions ($p < 0.05$), 0.45 *versus* 1.11 for HPV-6 associated external genital lesions ($p < 0.05$), 0.13 *versus* 0.52 for HPV-11 associated external genital lesions ($p < 0.05$), 0.52 *versus* 1.58 for HPV-6/11 associated condyloma acuminata ($p < 0.05$), and 3.61 *versus* 6.92 for HPV-6/11/16/18 persistent infection ($p < 0.05$)<br>No statistically significant reduction in HPV-16/18 associated external genital lesions or perineal epithelial neoplasia lesions
Palefsky *et al.* [35]	Randomized control trial<br>*N* = 602 (1:1 Ratio of subjects to Quadrivalent HPV Vaccine and Placebo Groups)<br>Age range: 16–26 years<br>Sex: Male<br>Other Inclusion Criteria: Men who have sex with men	All Subjects: Dose 1: month 0, Dose 2: month 2, Dose 3: month 6, Follow up: 7 months	Efficacy:<br>Against AIN associated with HPV infection of any type: 25.7% (95% CI = −1.1–45.6)<br>Against AIN associated with vaccine-type HPV infection: 50.3% (95% CI = 25.7–67.2)<br>Against grade 2+ AIN associated with vaccine-type HPV infection: 54.2% (95% CI = 18.0–75.3)<br>Against persistent HPV-6/11/16/18 infection: 59.4% (95% CI = 43.0–71.4)

HPV: human papillomavirus; *N*: number of subjects; CIN: cervical intraepithelial neoplasia; CI: confidence interval; OR: odds ratio; AIN: anal intraepithelial neoplasia.

Table S5. Phase I and II Bivalent HPV Vaccine Trials.

Phase	Name	Subject Characteristics	Follow up Duration	Results
I	HPV-002 [37]	*N* = 49 (Monovalent HPV-16 = 12, Monovalent HPV-18 = 12, Bivalent HPV-16/18 = 25)<br>Age range: 18–30 years<br>Sex: Female	All Subjects:<br>Dose 1: month 0,<br>Dose 2: month 1,<br>Follow up: 56 days<br>8 Subjects in Monovalent HPV-16 Group:<br>Dose 3: 112 days,<br>Follow up: 140 days	Safety:<br>No limiting toxicities or safety concerns<br>Immunogenicity:<br>Antibody and cell-mediated immune response generated<br>Bivalent vaccine generated immune response to both HPV-16 and HPV-18 comparable to the monovalent vaccines<br>Third dose of monovalent HPV-18 vaccine administered to 8 study subjects increased antibody levels in all cases
I/II	HPV-003 [37]	*N* = 61 (Bivalent HPV-16/18 = 31, Placebo = 30)<br>Age range: 18–30 years<br>Sex: Female	All Subjects:<br>Dose 1: month 0,<br>Dose 2: month 1,<br>Dose 3: month 6,<br>Follow up: 1 year	Safety:<br>Similar safety profiles found between bivalent vaccine and placebo group<br>No reported vaccine-related serious events<br>Immunogenicity:<br>Immune response to both HPV-16 and HPV-18 generated following second dose of vaccine<br>Increase in HPV-16/18 antibody levels after administration of third dose<br>Efficacy: No increase in the clearance rate of HPV-16/18 viral DNA in women with prior HPV-16 and/or HPV-18 infection

Table S5. *Cont.*

I/II	HPV-004 [37]	*N* = 60 (Bivalent with AS04 = 20, Bivalent with Aluminum Hydroxide = 20, Bivalent without Adjuvant = 20)<br>Age range: 18–30 years<br>Sex: Female	All Subjects:<br>Dose 1: month 0,<br>Dose 2: month 1,<br>Dose 3: month 6,<br>Follow up: 2 years	Safety:<br>No reported vaccine-related serious events<br>No significant difference in proportions of study subjects in the three groups with serious or systemic adverse events<br>Higher rate of local injection-site reactions with AS04 adjuvant<br>Immunogenicity:<br>Immune response to both HPV-16 and HPV-18 generated following second dose of vaccine<br>Increase in HPV-16/18 antibody levels after administration of third dose<br>Higher ELISA titers in the AS04 adjuvant group than the aluminum hydroxide or no adjuvant groups at day 210<br>Anti-HPV-16/18 antibodies following bivalent vaccine administration had kinetic profiles comparable to the neutralizing antibody profile
II	HPV-005 [37]	*N* = 209 (12 µg with AS04 = 59, 40 µg with AS04 = 64, 120 µg with AS04 = 59, 40 µg with Aluminum Hydroxide = 27)<br>Age range: 18–30 years<br>Sex: Female<br>Other Inclusion Criteria:<br>HPV-16/18 Negative at Baseline	All Subjects:<br>Dose 1: month 0,<br>Dose 2: month 1,<br>Dose 3: month 6,<br>Follow up:<br>360 days	Safety:<br>No reported vaccine-related serious events<br>All vaccine doses generally tolerated<br>Local injection-site reaction frequency proportional to increasing vaccine dose<br>Immunogenicity:<br>Immune response to both HPV-16 and HPV-18 generated following second dose of vaccine<br>Increase in HPV-16/18 antibody levels after administration of third dose<br>No significant effect of VLP dose of the AS04 formulations on the cellular immune response<br>ELISA titers of three VLP doses of the AS04 formulations suggested that the 12 µg dose may produce less of a humoral immune response

Table S5. *Cont.*

II	HPV-007 [38]	*N* = 1113 (Bivalent HPV-16/18 = 560, Placebo = 553)<br>Age range: 15–25 years<br>Sex: Female<br>Other Inclusion Criteria: HPV-16/18 Negative and Normal Cervical Cytology at Baseline	All Subjects:<br>Dose 1: month 0,<br>Dose 2: month 1,<br>Dose 3: month 6,<br>Follow up: 27 months<br>393 Subjects in Bivalent Vaccine Group and 383 Subjects in Placebo Group: Follow up: 4.5 years	Safety:<br>Similar safety profiles found between bivalent vaccine and placebo group<br>No reported vaccine-related serious events<br>Immunogenicity: Anti-HPV-16/18 antibody concentrations following vaccination remained 12-fold times greater than that achieved after natural infection<br>Efficacy:<br>Against HPV-16/18 infection incidence: 93.3% (95% CI = 87.4–98.7)<br>Against 12-month persistent infection: 100% (95% CI = 81.8–100)<br>Against HPV-16/18 associated CIN2+: 100% (95% CI = 51.3–100)<br>Against lesions independent of HPV DNA type: 71.9% (95% CI = 20.6–91.9)<br>Follow up: Vaccine is safe, immunogenic, and provides protection for up to 4.5 years[20] and 6.4 years[21]

HPV: human papillomavirus; *N*: number of subjects; CI: confidence interval; CIN: cervical intraepithelial neoplasia; PL: post-licensure.

Table S6. Phase III and Post-Licensure Bivalent HPV Vaccine Trials.

Phase	Name	Subject Characteristics	Follow up Duration	Results
III	PATRICIA [18]	Total Vaccine Group<br>*N* = 18,644 (Bivalent HPV-16/18 = 9319, Placebo = 9325)<br>Age range: 15–25 years<br>Sex: Female<br>Total Vaccine-naïve Group<br>*N* = 11,641 (Bivalent HPV-16/18 = 5822, Placebo = 5819)<br>Age range: 15–25 years<br>Sex: Female<br>Other Inclusion Criteria: Before sexual debut with no baseline evidence of HPV infection	All Subjects: Dose 1: month 0, Dose 2: month 1, Dose 3: month 6, Mean follow up: 40.9 months	Safety: Similar rates of serious adverse events, medical conditions, and new-onset chronic and autoimmune disease similar between bivalent vaccine and control groups<br>Efficacy:<br>Against HPV-16/18 associated CIN2+: 92.9% (96.1% CI = 79.9–98.3) in primary analysis and 98.1% (96.1% CI = 88.4–100) adjusted for probable causality in lesions with multiple oncogenic types<br>Against CIN2+ irrespective of HPV DNA type: 30.4% (96.1 CI = 16.4–42.1) in Total Vaccine Group and 70.2% (96.1% CI = 54.7–80.9) in Total Vaccine Group-naïve Group<br>Against CIN2+ associated with 12 non-vaccine oncogenic HPV types: 54.0% (96.1% CI = 34.0–68.4)
III	Hildesheim *et al*. [39]	*N* = 2189 (Bivalent HPV-16/18 = 1088, Placebo = 1101)<br>Age range: 18–25 years<br>Sex: Female<br>Other Inclusion Criteria: HPV DNA-positive at enrollment	All Subjects: Dose 1: month 0, Dose 2: month 1, Dose 3: month 6, Follow up: 6 months	Efficacy: No evidence of accelerated viral clearance in HPV-positive women at 6 or 12 months<br>Vaccine efficacy of viral clearance at 6 months compared to placebo of 2.5% (95% CI = −9.8–13.5)<br>Vaccine efficacy of viral clearance at 12 months compared to placebo of −2.09% (95% CI = −24.3–16.3)

Table S6. *Cont.*

III	Pedersen *et al.* [40]	*N* = 773 (10–14 Years of Age = 158, 15–25 Years of Age = 458) Age range: 10–25 years Sex: Female	All Subjects: Dose 1: month 0, Dose 2: month 1, Dose 3: month 6, Follow up: 7 months	Safety: No difference in safety profile of vaccine between two age groups Immunogenicity: Seroconversion similar between two age groups Antibody titers approximately twice as high in females aged 10–14 years compared to females aged 15–25 years
PL	Angelo *et al.* [42]	Post-licensure passive surveillance safety data from global pharmacovigilance	Data from May 18, 2007–November 17, 2011	Safety: Safety profile and reported adverse events comparable to pre-licensure data No evidence to support post-vaccine immune-mediated disease

HPV: human papillomavirus; *N*: number of subjects; CI: confidence interval; CIN: cervical intraepithelial neoplasia; PL: post-licensure.

© 2015 by the authors; licensee MDPI, Basel, Switzerland. This article is an open access article distributed under the terms and conditions of the Creative Commons Attribution license (http://creativecommons.org/licenses/by/4.0/).
